# Natural Killer Cells Are Characterized by the Concomitantly Increased Interferon-γ and Cytotoxicity in Acute Resolved Hepatitis B Patients

**DOI:** 10.1371/journal.pone.0049135

**Published:** 2012-11-01

**Authors:** Juanjuan Zhao, Yonggang Li, Lei Jin, Shuye Zhang, Rong Fan, Yanling Sun, Chunbao Zhou, Qinghua Shang, Wengang Li, Zheng Zhang, Fu-Sheng Wang

**Affiliations:** 1 Center for Infection and Immunity, Institute of Biophysics, Chinese Academy of Science, Beijing, China; 2 Integrative Medicine Center, Beijing 302 Hospital, Beijing, China; 3 Research Center for Biological Therapy, Institute of Translational Hepatology, Beijing 302 Hospital, Beijing, China; 4 Department of Liver Disease, Tai-An 88 Hospital, Tai-An, Shan Dong Province, China; Karolinska Institutet, Sweden

## Abstract

Natural killer (NK) cells are abundant in the liver and have been implicated in inducing hepatocellular damage in patients with chronic hepatitis B virus (HBV) infection. However, the role of NK cells in acute HBV infection remains to be elucidated. We comprehensively characterized NK cells and investigated their roles in HBV clearance and liver pathology in 19 chronic hepatitis B (CHB) patients and 21 acute hepatitis B (AHB) patients as well as 16 healthy subjects. It was found that NKp46^+^ NK cells were enriched in the livers of AHB and CHB patients. We further found that peripheral NK cells from AHB patients expressed higher levels of activation receptors and lower levels of inhibitory receptors than those from CHB patients and HC subjects, thus displaying the increased cytolytic activity and interferon-γ production. NK cell activation levels were also correlated positively with serum alanine aminotransferase levels and negatively with plasma HBV DNA levels in AHB patients, which is further confirmed by the longitudinal follow-up of AHB patients. Serum pro-inflammatory cytokine and chemokine levels were also increased in AHB patients as compared with CHB and HC subjects. Thus, the concomitantly increased interferon-γ and cytotoxicity of NK cells were associated with liver injury and viral clearance in AHB patients.

## Introduction

Hepatitis B virus (HBV) infection is a major human threat that affects approximately 400 million people worldwide. While HBV infection in utero or early in life results in chronic infection, adults infected with this virus usually develop an acute self-limited infection [1]. Particularly, acute hepatitis B (AHB) is difficult to diagnose in the clinic because 90% of adult patients enter the convalescence period without obvious clinical manifestations by the time the patient first presents to the physician. Therefore, little is known about the early events in acute HBV infection.

The host immune response is generally considered to drive disease progression of HBV infection [1]; however, the relevant immunological factors that determine different outcomes of HBV infection are unknown [2], [3]. Generally, HBV-specific T cell responses are thought to be of considerable importance in viral control and immune-mediated liver damage [4]. During acute HBV infection, virus-specific T cell responses are often readily detectable and multi-specific [5]–[7]; while in chronic HBV infections, virus-specific T cell responses are generally weak and display functional exhaustion as a result of the upregulation of programmed death-1 [8], [9], T cell attrition through Bcl2 signaling [10] and impaired T cell receptor signaling through the ζ-chain [11]. Despite the associations between the adaptive T cell responses, viral clearance and liver damage during acute and chronic HBV infection, the innate immune effector mechanisms that are responsible for viral clearance and liver pathogenesis remained obscure [12].

Innate immune cells, for example natural killer (NK) cells, are predominantly enriched into the human liver [13], [14]. NK cell activation is generally regulated by a set of activation and inhibitory receptors that are expressed on the cell surface [15]. The intensity and quality of NK cell responses also depend on the cytokine microenvironment. Type I interferon (IFN), interleukin (IL)-12, IL-15 and IL-18 are potent activators of NK cell function [16]. Several recent studies have reported the role of NK cells in liver injury in chronic Hepatitis C virus (HCV) [17], [18] and HBV infections [18]–[21], in which NK cells are biased towards cytolytic activity but without a concomitant increase in IFN-γ production. In contrast, in acute HBV infection, early NK cell responses are likely to contribute to the initial control of infection and to allow timely development of an efficient adaptive immune response [7], [22]. However, this early activation and IFN-γ production by NK cells can be transiently inhibited by a surge of IL-10 during peak viremia [23]. Although NK cells are known to be functionally involved in liver pathogenesis in chronic HBV-infected patients [18]–[21], limited data are available regarding the immune status and clinical significance of these cells in acute HBV infected patients with longitudinal follow-up.

This study comprehensively characterized NK cells in a cohort of acute HBV-infected patients with longitudinal follow-up, and found that increased IFN-γ production by NK cells may play a role in HBV control, and the increased cytolytic capacity of these cells may promote liver injury. Our findings may facilitate the rational development of immunotherapeutic strategies to enhance viral control while limiting or abolishing liver injury in HBV infection.

## Results

### Increased CD56^bright^ NK subsets, but reduced CD56^dim^ NK subsets in patients with acute and chronic HBV infection

**Figure 1:**
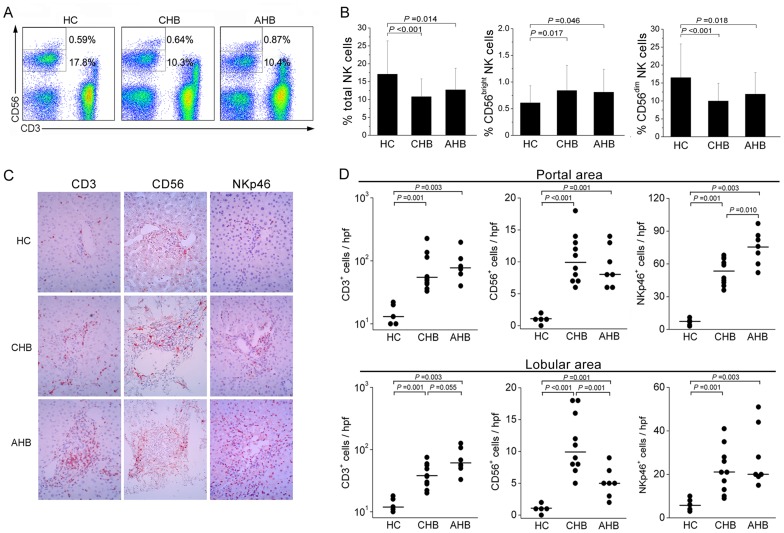
Increased CD56^bright^ NK subsets in patients with acute and chronic HBV infection. (A) Flow cytometric analysis of CD3 and CD56 expression by peripheral blood mononuclear cells (PBMCs) after application of a lymphocyte gate. Values indicate the percentages of CD3^−^CD56^bright^ and CD3^−^CD56^dim^ NK cell subset among lymphocytes. (B) Pooled data showing the frequencies of total NK, CD56^bright^ and CD56^dim^ NK cells in HC subjects (n = 16) and CHB (n = 19) and AHB patients (n = 21). The data represent the mean ± SD. *P*-values are shown. (C) Immunohistochemical detection of CD3^+^, CD56^+^ and NKp46^+^ cells in the liver tissues of CHB (n = 10) and AHB patients (n = 7) and HC donors (n = 5) (magnification, ×400). Positively stained cells appear red and were present in both portal and lobular areas in the livers. (D) Pooled data showing CD3^+^, CD56^+^ and NKp46^+^ cell counts in both portal and lobular areas in CHB (n = 10) and AHB patients (n = 7) and HC donors (n = 5). Each dot represents one subject. Horizontal lines illustrate the median percentiles. *P*-values are shown. HC, healthy controls; CHB, chronic hepatitis B; AHB, acute hepatitis B. (B and D), Multiple comparisons were first made among the three groups using the Kruskal-Wallis *H* non-parametric test. Then the comparisons between two groups were performed using the Mann-Whitney *U* test.

Analysis of the frequencies of circulating total NK cells (CD3^−^CD56^+^) and NK subsets (CD3^−^CD56^bright^ and CD3^−^CD56^dim^) in all enrolled subjects (Figure 1A) revealed that the total NK cell frequencies were significantly decreased in both CHB and AHB patients compared with those in HC subjects (both *P*<0.05; Figure 1B). The contribution of the two NK subsets to the total NK cell frequency was investigated in these individuals. It was observed that CD3^−^CD56^bright^ NK cells were significantly expanded in both CHB and AHB patients compared to the HC group. In contrast, CD3^−^CD56^dim^ NK cells were greatly reduced in CHB and AHB patients compared with the HC group (Figure 1B). We further analyzed the correlation between the frequencies of total NK cells and NK cell subsets and clinical parameters such as viral load and ALT levels in AHB patients. There were no significant associations between the frequencies of NK cell subsets with HBV load or serum ALT levels (data not shown). These data indicate a re-distribution in peripheral NK cell compartments in patients with acute and chronic HBV infections.

### NK cells were enriched in patients with acute and chronic HBV infections

The distribution of hepatic CD3^+^, CD56^+^ and NKp46^+^ (a unique NK marker) cells were investigated by immunohistochemical staining (Figure 1C). Few CD3^+^, CD56^+^ and NKp46^+^ cells were present in the livers of healthy donors. In contrast, numerous CD3^+^, CD56^+^ and NKp46^+^ cells were frequently seen in the livers of CHB and AHB patients. Quantitative analysis of hepatic CD3^+^, CD56^+^ and NKp46^+^ cell counts in both portal and lobular areas further confirmed this observation (Figure 1D). Notably, more CD56^+^ and NKp46^+^ cells were found to be accumulated in the moderately and severely inflamed lobular areas of liver in AHB patients, where hepatocyte necrosis is usually observed. These data indicate that NK cells were also enriched in the livers of AHB and CHB patients.

### AHB patients were characterized by the increased activation of both NK and T cells in peripheral blood

**Figure 2:**
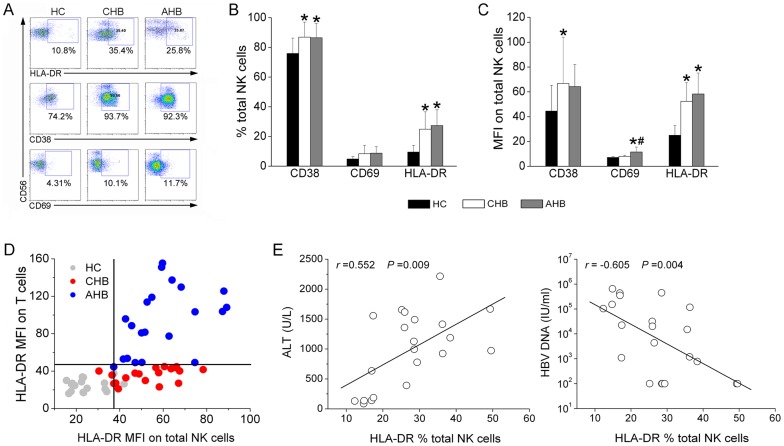
NK cells displayed an increased activation status in AHB patients in vivo. (A) Representative dot plots depict the expression of activation markers CD69, CD38 and HLA-DR on total NK cells among HC subjects and CHB and AHB patients. CD3^−^CD56^+^ NK cells were gated. Values in quadrants represent the percentages of CD3^−^CD56^+^ NK cells that express activation markers. (B and C) Pooled data showed the percentages and MFI of CD69, CD38 and HLA-DR expression by total NK cells from the HC (n = 16), CHB (n = 19) and AHB (n = 21) groups. Multiple comparisons were first made among the three groups using the Kruskal-Wallis *H* non-parametric test. Then the comparisons between two groups were performed using the Mann-Whitney *U* test. *P<0.05 compared with HC subjects; ^#^ *P*<0.05 compared with CHB patients. (D) Scatter plots show the HLA-DR MFI on NK cells and T cells simultaneously in HC (n = 16), CHB (n = 19) and AHB (n = 21) subject groups. (E) Correlation analysis between HLA-DR percentages on total NK cells and plasma ALT and HBV DNA levels in AHB patients (n = 21). Correlations between the two variables were evaluated using the Spearman rank correlation test. *r*, correlation coefficient; *P*-values are shown.

Expression of CD69, CD38 and HLA-DR was analyzed to assess the activation state of NK cells and conventional T cells in CHB and AHB patients (Figure 2A). It was observed that the frequencies of activation markers, CD38 and HLA-DR but not CD69 were significantly increased on NK cells in both AHB and CHB patients compared with HC subjects (Figure 2B). A similar pattern was observed for the MFI of CD38, CD69 and HLA-DR expression on total NK cells (Figure 2C). Interestingly, the expression levels of these activation markers on total T cells were also significantly increased in AHB patients but not in CHB patients as compared with that in HC subjects (data not shown). The further investigation demonstrate a clear discrimination between these three groups of subjects on the basis of HLA-DR MFI levels: HC, NK^low^ T^low^; CHB: NK^high^ T^low^ and AHB: NK^high^ T^high^ (Figure 2D). Correlation analysis further confirmed that the percentage HLA-DR expression on total NK cells correlated positively with serum ALT levels and negatively with HBV DNA levels (both *P*<0.05; Figure 2E). These data suggest that the presence of activated NK cells is closely associated with liver necroinflammation and HBV clearance in AHB patients.

### NK cells displayed abnormal NK receptor expression in AHB patients

**Figure 3:**
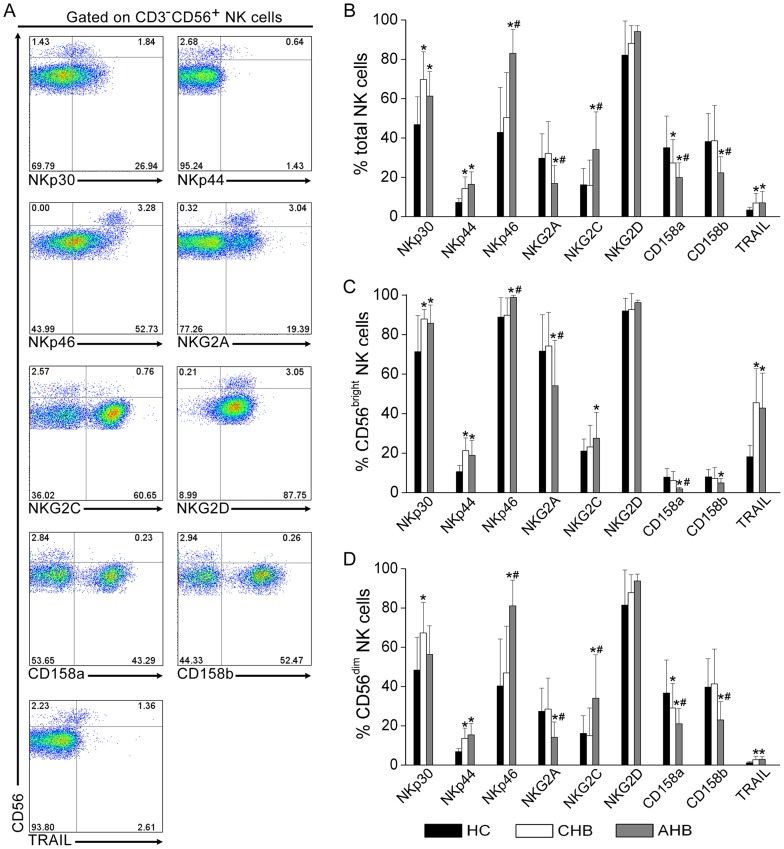
NK cells displayed abnormal NK receptor expression in AHB patients. (A) Representative dot plots depict the expression of NK activation receptors (NKp30, NKp44, NKp46, NKG2D and NKG2C) and inhibitory receptors (CD158a, CD158b and NKG2A) and TRAIL in an AHB patient. CD3^−^CD56^+^ NK cells were gated. Values in quadrants represent the percentages of CD3^−^CD56^+^ NK cells that express NK receptors. (B–D) Pooled data show the frequencies of total (B), CD56^bright^ (C) and CD56^dim^ (D) NK cell subsets expressing NK activation receptors (NKp30, NKp44, NKp46, NKG2D and NKG2C) and inhibitory receptors (CD158a, CD158b and NKG2A) and TRAIL in HC (n = 16), CHB (n = 19) and AHB (n = 21) groups. Multiple comparisons were first made among the three groups using the Kruskal-Wallis *H* non-parametric test. Then the comparisons between two groups were performed using the Mann-Whitney *U* test. The data represent the mean ± SD. * *P*<0.05 compared with HC subjects; ^#^ *P*<0.05 compared with CHB patients.

We further analyzed NK cell receptor expression including activation receptors NKp30, NKp44 and NKp46, NKG2D and NKG2C and inhibitory receptors NKG2A, CD158a and CD158b, as well as TRAIL expression which has been demonstrated previously to be up-regulated on NK cells in CHB patients [20], [21] in the three groups (Figure 3A). A significant upregulation of activation receptors NKp30, NKp44, NKp46, NKG2C but not NKG2D was observed on total NK cells (Figure 3B) as well as CD56^bright^ (Figure 3C) and CD56^dim^ (Figure 3D) subsets in AHB and CHB patients as compared with HC subjects. A similar increase of TRAIL on NK subsets was also observed in AHB and CHB patinets as compared with HC subjects (Figure 3B–3D). However, the inhibitory receptors CD158a and CD158b as well as NKG2A on total and NK subsets were significantly down-regulated in HBV infected individuals compared with HC subjects. Interestingly, it was observed that NKp46 and NKG2C expression levels were further upregulated by peripheral NK subsets in AHB patients compared with CHB subjects, and inhibitory receptors CD158a, CD158b and NKG2A were further downregulated on NK subsets in AHB patients compared with CHB patients (Figure 3B–3D). Thus, activation receptor-expressing NK cells were preferentially enriched in AHB patients, while the inhibitory receptor-expressing NK cells were further reduced in AHB patients compared with CHB patients.

### NK cells from AHB patients displayed the increased cytolytic activity and IFN-γ responses

**Figure 4:**
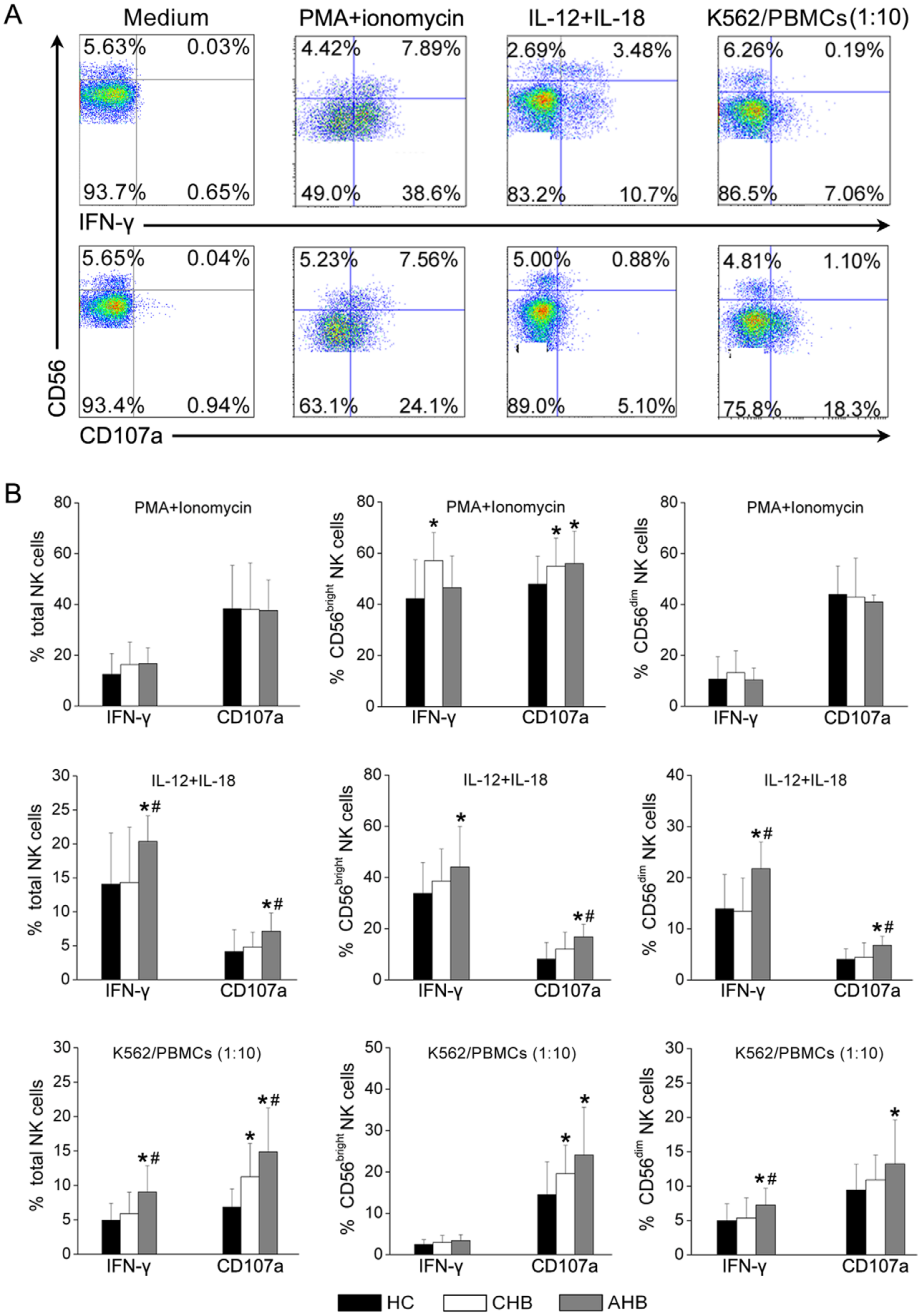
IFN-γ production and CD107a expression by NK cells were concurrently increased in AHB patients. (A) Representative dot plots depict IFN-γ and CD107a expression by NK subsets from an HC subject following PMA/ionomycin, IL-12/IL-18 and K562 stimulation. CD3^−^CD56^+^ cells were gated. Values in the quadrants represent the percentages of CD3^−^CD56^+^ NK cells that express IFN-γ or CD107a. (B) Pooled data show the proportion of IFN-γ^+^ and CD107a^+^ NK cell subsets in response to three stimulation conditions in HC (n = 16), CHB (n = 19) and AHB (n = 16) groups. Multiple comparisons were first made among the three groups using the Kruskal-Wallis *H* non-parametric test. Then the comparisons between two groups were performed using the Mann-Whitney *U* test. The data represent the mean ± SD. *P<0.05 compared with HC subjects; ^#^ *P*<0.05 compared with CHB patients.

The functional properties of NK cell subsets were investigated by monitoring intracellular IFN-γ production and CD107a expression in response to major histocompatibility complex (MHC)-devoid target cells (K562 cells), cytokine IL-12 and IL-18 and mitogenic PMA/ionomycin stimulation (Figure 4A). It was observed that both CD56^bright^ and CD56^dim^ NK cells responded to stimulation with PMA/ionomycin, IL-12/IL18 and MHC-devoid K562 cells. Differently, PMA/ionomycin induced high levels of IFN-γ and CD107a in both CD56^bright^ and CD56^dim^ NK cell subsets. IL-12/IL-18 induced high and median levels of IFN-γ production by CD56^bright^ and CD56^dim^ NK cell subsets respectively, but induced low levels of CD107a in both subsets. However, following stimulation by MHC-devoid K562 cells, CD107a expression was upregulated in the CD56^dim^ NK cell subset but not in the CD56^bright^ NK cell subset. IFN-γ was produced by both CD56^bright^ and CD56^dim^ NK cell subsets at low levels (Figure 4A).

IFN-γ and CD107a production was then compared in total NK cells, CD56^bright^ and CD56^dim^ NK cell subsets among the three groups. The ability of total NK cells and CD56^dim^ NK cells to produce both IFN-γ and CD107a expression in response to PMA/ionomycin was similar in the three groups of subjects. In contrast, CD56^bright^ NK cells have more potential to produce IFN-γ and CD107a in AHB and CHB patients in response to PMA/ionomycin stimulation, as compared with those from HC subjects (Figure 4B).

Notably, the expression of IFN-γ and CD107a by total NK was significantly elevated in AHB patients as compared with CHB patients and HC subjects following the stimulation with IL-12/IL-18 and K562 cells respectively (Figure 4B). Furthermore, CD107a expression by the CD56^bright^ subset and both IFN-γ and CD107a expression by the CD56^dim^ NK cell subset were also significantly increased in response to IL-12/IL-18 in AHB patients. IFN-γ production by the CD56^dim^ NK cell subset was also significantly increased in response to K562 cells in AHB patients. No significant differences in IFN-γ and CD107a expression by NK subsets were detected between CHB patients and HC subjects with the exception of CD107a expression by total NK cells and the CD56^bright^ subset, which was higher in CHB patients compared with HC subjects. These data suggest that the increased NK cell activity indicated by IFN-γ and CD107a upregulation in AHB patients compared with CHB patients and HC subjects.

### Increased levels of plasma pro-inflammatory cytokines and chemokines in AHB patients

**Figure 5:**
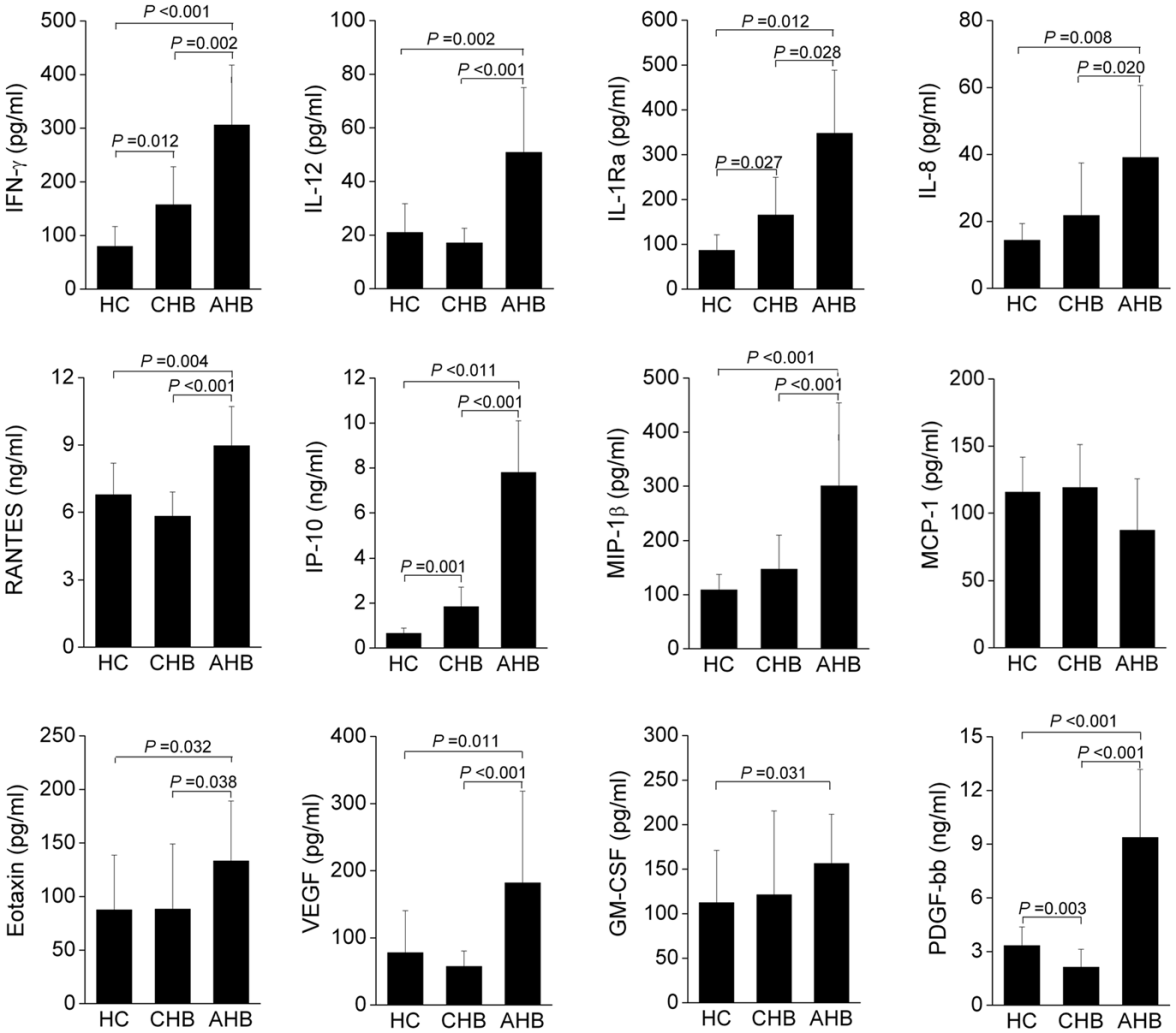
Plasma cytokine levels were significantly increased in AHB patients. Plasma cytokine levels including inflammatory cytokines IL-12p70, IL-1Ra and IFN-γ, chemokines including RANTES, IP-10, MIP-1β, MCP-1, IL-8 and eotaxin, and growth factors including GM-CSF, VEGF and PDGF-bb in HC (n = 10), CHB (n = 10) and AHB (n = 10) groups are shown. Multiple comparisons were first made among the three groups using the Kruskal-Wallis *H* non-parametric test. Then the comparisons between two groups were performed using the Mann-Whitney *U* test. The data represent the mean ± SD. *P*-values are shown.

Plasma levels of the pro-inflammatory cytokines (IL-1Ra, IL-12p70 and IFN-γ), chemokines (IL-8, RANTES, IP-10, MIP-1β and eotaxin) and growth factors (VEGF, GM-CSF and PDGF-BB) were significantly increased in AHB patients compared with those of CHB patients and HC subjects (Figure 5). No significant changes of these markers were observed between CHB patients and HC subjects, with the exception of IFN-γ, IL-1ra, IP-10 and PDGF-bb, which were also increased in CHB patients compared with HC subjects (Figure 5). Other cytokine levels in plasma including IL-1β, IL-2, IL-4, IL-5, IL-6, IL-7, IL-8, IL-9, IL-10, IL-13, IL-15, IL-17, basic FGF, G-CSF, MIP-1α, and TNF-α were not compared among these three groups due to undetectable levels. These data indicate higher levels of systemic inflammation in AHB patients compared with CHB patients.

### Longitudinal detection of NK cell activation in AHB patients

**Figure 6:**
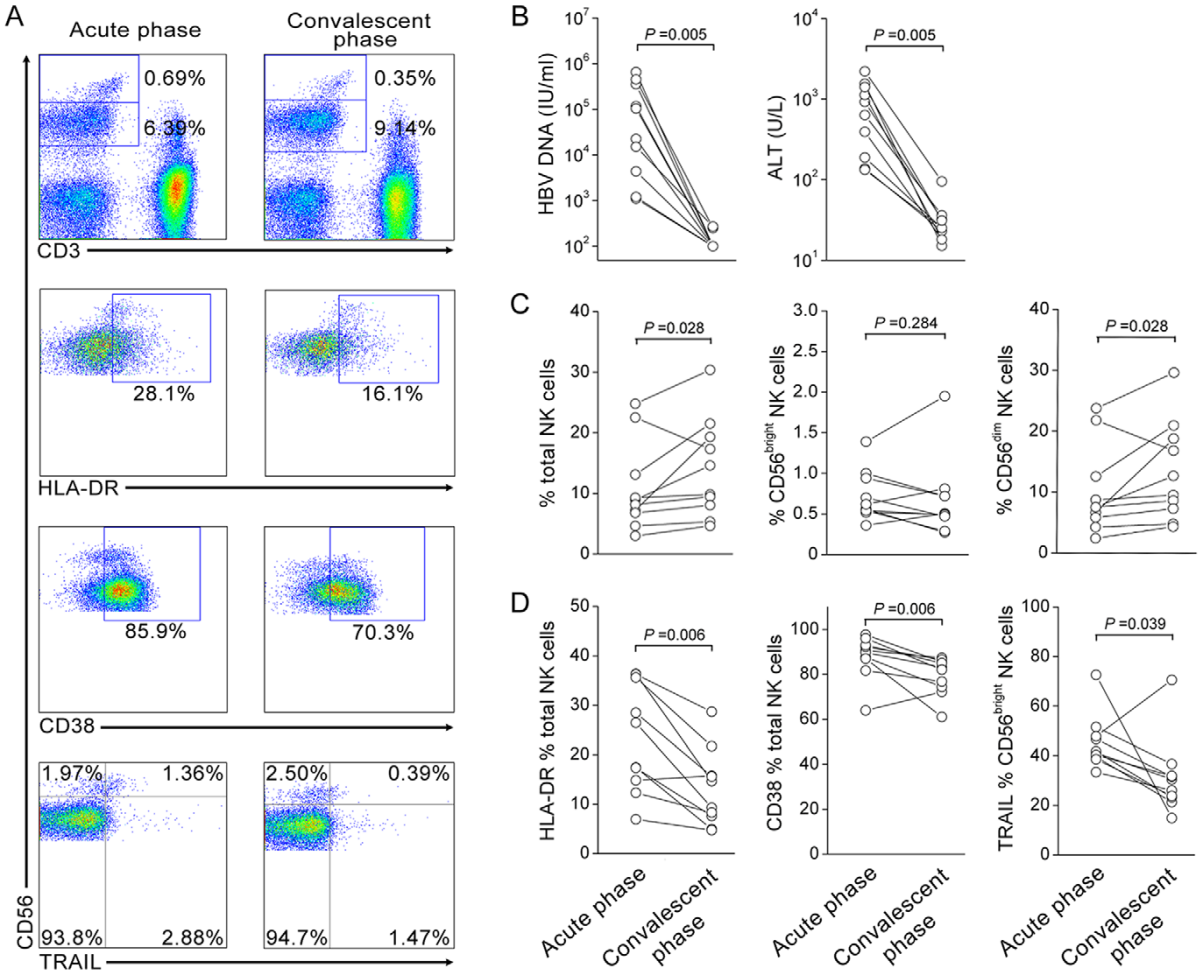
Dynamic expression of clinical and immunological parameters in AHB patients (n = 10). (A) The representative dot plots indicate the NK subsets frequencies and activation markers HLA-DR and CD38 as well as TRAIL expression on NK cells in AHB patients at acute and convalescent phases of illness. Values indicate the percentages of HLA-DR, CD38 and TRAIL among CD3^−^CD56^+^ NK cells. (B–D) The dynamic change of HBV DNA and serum ALT levels (B), the percentages of total, CD56^bright^ and CD56^dim^ NK cells (C) and HLA-DR, CD38 and TRAIL expression on NK cell subsets (D) were analyzed in AHB patients at the acute and convalescent phases of illness, respectively. *P* values are shown. The comparisons were performed using the Wilcoxon matched pairs *T* test.

The plasma ALT and HBV load and NK cell frequency, activation markers HLA-DR and CD38 and TRAIL expression were longitudinally detected at acute (median 2 weeks since the clinical onset, range [1 week–3 weeks]) and convalescence phases (median 15 weeks since the clinical onset, range [5 weeks–27 weeks]) in 10 of AHB patients (Figure 6A). It was found that the serum HBV loads and ALT levels were both reduced to undetectable levels or normal levels (<40 U/L) from the acute phase to convalescence phase in these AHB patients (Figure 6B). Interestingly, the percentages of total NK cells and CD3^−^CD56^dim^ NK cells were slightly increased at convalescence phase as compared with acute phase in these AHB patients; in contrast, the CD3^−^CD56^bright^ frequency remained stable during the process (Figure 6C). More important, we observed that NK cell activation indicated by HLA-DR and CD38 expression as well as TRAIL expression were all significantly reduced at convalescence phase as compared with acute phase in these AHB patients (Figure 6D). These data indicated that NK cell activation was decreased with the disease recovery in AHB patients.

## Discussion

The current study characterized NK cell subsets during the early and convalescence phase of in a cohort of AHB patients and demonstrated that NK cells are preferentially activated at the acute phase but decreased at the convalescence phase, thus associating with both liver injury and simultaneous viral clearance. These findings clearly demonstrated the immune status of NK cells in vivo and defined the double-edged roles of NK cells in viral clearance and liver injury in patients with acute HBV infection.

Similar to NK cells from immune activation (IA) patients with chronic HBV infection [20], AHB patients exhibited decreased total NK frequencies and increased NK activation status. Interestingly, NK cells from AHB patients displayed several unique properties that differed from those of CHB patients. NK cells in AHB patients displayed a greater potential to produce IFN-γ and CD107a compared with that in CHB patients. The increased IFN-γ may associate with HBV clearance via a non-cytolytic mechanism [24], [25]; while the increased CD107a expression by NK cells may be associated with hepatocyte injury in AHB patients. In contrast, as described in our previous report [20], NK cells exhibited an enhanced cytolytic activity without concomitant IFN-γ production, which may result in liver damage but favor viral persistence in CHB patients. TRAIL up-regulation may further aggravate the liver injury as like in CHB patients [21]. The role of NK cells in viral clearance in acute HBV infection was also supported by several previous reports in which early large quantities of IFN-γ production by NK cells may contribute to the initial control of infection and allow timely development of an adaptive immune response [7], [22]. These findings, in combination with recent studies of CHB patients [18] and chronic HCV infection [17], support the concept that the enhanced NK cytolytic activity may mediate liver injury in acute and chronic hepatitis virus infection, whereas sufficient IFN-γ production by NK cells may also be sufficient to achieve viral clearance in acute viral infection. In particular, NKp46^+^ NK cells were enriched in the livers of AHB and CHB patients may further strengthen their antiviral and hepatocytic effects because several recent studies have identified that NKp46^+^ NK cells were characterized by both IFN-γ production and high cytolytic activity and have the potential to control HCV replication and to kill hepatic stellate cells [26]–[28]. Their impact on the liver disease progression in HBV infection will be of interest.

The mechanisms underlying the differential regulation of NK activity in AHB and CHB patients remain to be elucidated but may include variation in expression profiles of NK receptor/ligands and cytokines [29]. In this regard, the present study showed that there are more types of activation receptors were upregulated (such as NKp46, NKG2C) and more types of inhibitory receptors (CD158a, CD158b and NKG2A) were downregulated by NK cells from AHB patients compared with those from CHB patients. Furthermore, the expression levels of the activation receptors NKp46 and NKG2C were significantly higher and those of inhibitory receptors CD158a, CD158b and NKG2A were significantly lower in NK cell subsets from AHB patients compared with those from CHB patients. This significant bias in activation and inhibitory receptor expression may partially explain the increased activity of NK cells in AHB patients compared with CHB patients. Furthermore, expression profiles of cytokines such as IFN-α and IL-12/IL-15/IL-18 may also be involved in the regulation of NK cell activity in chronic HCV and HBV infection [20], [30]. In this study, significantly increased plasma levels of multiple cytokines, including IL-12 and chemokines, including MIP-1β, IP-10, IL-8, RANTES and eotaxin as well as the growth factors GM-CSF, PDGF-BB and VEGF in AHB patients suggested an increased systematic inflammation in AHB patients. Further studies are required to elucidate the regulatory roles of these altered cytokine and chemokine profiles on NK cell activity in AHB patients.

Another important finding of this study was the activation of both NK cells and T cells in AHB patients, while in contrast only NK cells were activated and T cells deactivated in CHB patients. Previous studies have shown that T cells are often readily activated and multi-clonal in acute HBV infection [5]–[7], [31]. However, in chronic HBV infection, virus-specific T cell responses are generally weak and exhibit functional exhaustion, also indicated by deactivation of T cells. Thus, the activation of both innate immunity and adaptive immunity may favor viral clearance [32]. However, activation of innate immunity such as NK cell activation mediates relatively poor viral clearance, but has the potential to mediate liver injury. This notion was further confirmed in longitudinal detection of NK cells in AHB patients who displayed a decrease in NK cell activation and an increase in NK cell percentages at the convalescence phase as compared with that at acute phase; this process was accompained by HBV clearance and the recovery of liver injury in these patinets. This finding also suggested that NK activation combined with T cell activation serves as a marker to distinguish acute hepatitis and the acute exacerbation of chronic hepatitis, although this requires confirmation in further prospective studies.

This study was limited by the lack of access to samples from the incubation phase of acute HBV infection. During this initial phase after viral entry, there appears to be a temporary block to HBV replication and spread [33]. It can be speculated that this is partially mediated by innate immune mechanisms, as suggested by elevated IL-15 and NK activation/function just before the expansion phase in some patients [23]. Another study also showed activation of NK and NKT cells within 72 hours of experimental infection with woodchuck hepadnavirus, resulting in transient suppression of viral replication [34]. Another limitation in our study was the absence of the detection of NK cell function in the intrahepatic compartment, which is the site of viral replication, although NK cell distribution was just detectable in liver biopsies through IHC staining using our previously stored samples [35]. However, changes in the circulation have been found to closely mirror those in the liver in acute HBV infection in chimpanzees [36]. In accordance with this, we have previously demonstrated NK activation in the liver of patients with chronic HBV infection [20].

In conclusion, this study indicates that NK cells are activated and exhibit concomitantly increased cytolytic activity and IFN-γ production in the acute phase, which was subsequently decreased at the the convalescence phase as companied by the HBV clearance and the recovery of liver injury in these AHB patinets. This study, therefore, highlights the roles of NK cells in liver immunopathogenesis in acute HBV infection and will facilitate the rational development of immunotherapeutic strategies that decrease NK cytolytic capacity while enhancing IFN-γ production in chronic HBV infection.

## Materials and Methods

### Ethics Statement

The study protocol was approved by the Ethics Committee of Beijing 302 Hospital. The individual in this manuscript has given written informed consent (as outlined in the PLoS consent form) to publish these case details.

### Study subjects

Twenty-one AHB and 19 CHB patients were recruited into this study. All patients were diagnosed according to previously described criteria [20], [35] and had not received antiviral therapy or immunosuppressive drugs within 6 months of the start of sampling. Sixteen age- and sex-matched healthy individuals were enrolled as controls (HC). Individuals with concurrent HCV, human immunodeficiency virus infections, autoimmune liver diseases or alcoholic liver disease were excluded. All of these subjects enrolled in the study are hepatitis D virus negative except one CHB patient who displayed HDV-specific IgG and antigen positive. The basic characteristics of these enrolled subjects are listed in Table 1.

**Table 1:** Characteristics of subjects at the sampling time in the study.

	HC	CHB	AHB
Case	16	19	21
Age	30 (25–45)	38 (22–65)	37 (17–56)
Gender (M/F)	12/4	15/4	15/6
ALT (U/L)	NA	242 (42–1298)	974 (88–2216)*
HBV DNA (IU/ml)	NA	1.5×10^7^ (4.94×10^2^–4.17×10^8^)	2.01×10^4^ (<100–6.51×10^5^)*

Data are shown as median and range. * *P*<0.05 versus CHB patients; HC: healthy controls; CHB, chronic hepatitis B; AHB, acute hepatitis B. NA: not applicable.

For AHB patients, alanine aminotransferase (ALT) levels at clinical onset were at least 10-fold greater than the upper limit of the normal level and first detection of serum hepatitis B surface antigen (HBsAg) and immunoglobulin (Ig) M anti-hepatitis B core antigen (HBcAg) [5], [6], [35]. The detailed clinical descriptions of acute phase at the onset of sampling were shown in the Table 2. Among these 21 AHB patients, 10 of patients were also followed-up for immunological detection at the convalescent phases.

**Table 2:** The baseline clinical data of the enrolled AHB patients.

Pt No	Sampling weeks since clinical onset	Age (years)	Gender	sAg/sAb/eAg/eAb/cAb	ALT (U/L)	HBV DNA (IU/ml)
1	1w	19	male	+/−/+/+/+	1415	1200
2	3w	51	female	+/−/−/+/+	1188	779
3	1w	35	male	+/+/−/+/+	974	100
4	1w	34	male	−/+/−/+/+	132	104000
5	2w	17	female	+/−/+/+/+	1617	20100
6	2w	19	male	+/−/+/−/+	2216	15100
7	1w	36	male	+/−/+/−/+	1491	100
8	1w	26	female	+/−/+/+/+	1656	100
9	2w	43	male	+/−/+/−/+	1669	100
10	3w	37	male	+/−/−/+/+	141	436000
11	3w	37	male	−/+/−/+/+	88	153000
12	2w	30	male	+/−/+/+/+	1360	31800
13	3w	46	male	+/−/+/+/+	779	100
14	3w	38	male	+/+/−/+/+	635	353000
15	1w	56	female	−/−/−/+/+	997	100
16	1w	38	male	+/+/−/+/+	1557	1100
17	1w	26	female	+/−/+/−/+	1128	449000
18	3w	39	male	−/−/−/−/+	188	22400
19	3w	39	male	−/−/−/+/+	137	651000
20	2w	43	female	+/−/−/+/+	392	4380
21	2w	49	male	+/−/−/−/+	925	117000

Peripheral blood mononuclear cells (PBMCs) were isolated from all enrolled subjects. Liver biopsies were collected from AHB patients (n = 7), CHB patients (n = 10) and healthy donors (n = 5), which have been used in our previous studies [20], [35]. These healthy liver tissue samples, were obtained from the healthy donors whose livers were used for transplantation after receiving the informed consent from each donor. For pathological evaluation, liver biopsy specimens were embedded in Tissue Tek for in situ immunohistochemical staining.

### FACS analysis

All antibodies were purchased from BD Biosciences (San Jose, CA, USA) with the exception of phycoerythrin (PE)-conjugated anti-NKG2A, anti-NKG2C, anti-NKG2D, anti-NKp30 and anti-NKp46 antibodies, which were purchased from R&D Systems (Minneapolis, MN). Peripheral NK subset frequencies and NK receptor expression were analyzed according to previously described protocols [20], [37]. For detection of NK cell activation, PBMCs were incubated with PE-conjugated anti-CD3, APC-conjugated anti-CD56 and PerCP-conjugated anti-HLA-DR, or FITC-conjugated anti-CD69 or FITC-conjugated anti-CD38. For intracellular IFN-γ and CD107a staining, cells were permeabilized and stained with the corresponding intracellular antibodies. Cells were then analyzed using FACSCalibur and Flowjo software (TreeStar, Ashand, OR, USA). At least 200,000 events were acquired per run.

### Degranulation of NK cells and IFN-γ detection

CD107a degranulation is now widely used to assess NK cell cytotoxic potential [35] Briefly, freshly isolated PBMCs (5×10^5^) were directly stimulated with PMA (100 ng mL-1) and ionomycin (1 µg mL-1), IL-12 (10 ng mL-1) in combination with IL-18 (10 ng mL-1; Biovision, PA, CA) or K562 cells at an effector to target ratio (E:T) of 10∶1. Unstimulated PBMCs served as negative controls. Anti-CD107a and GolgiStop were added directly into the medium and incubated for 5 h. Cells were then collected and stained with surface antibodies and stained intracellularly with anti-IFN-γ.

### Immunohistochemical staining

Paraform-fixed liver biopsy sections (5 μm) were incubated with anti-CD3 (clone F7.2.38, Dako Company, Denmark), anti-CD56 (clone 123C3, Dako Company) antibodies and anti-NKp46 (clone 195314, R&D Systems) overnight at 4°C, respectively, after blocking endogenous peroxidase activity with 0.3% H_2_O_2_. 3-Amino-9-ethyl-carbazole (red color) was used for single staining. High powered fields (hpf, ×400) were used for counting positive cells according to previously described protocols [20], [35]. Positive cells were counted in three different fields by two independent observers. Results are expressed as the median and range of all tested patients in each group.

### Luminex

Plasma cytokines were detected using a Luminex Bio-Plex ProTm Human Cytokine Standard Group I 27-Plex kit (Cat No: 5021099, including IL-1β, IL-1ra, IL-2, IL-4, IL-5, IL-6, IL-7, IL-8, IL-9, IL-10, IL-12p70, IL-13, IL-15, IL-17, Eotaxin, basic FGF, G-CSF, GM-CSF, IFN-γ, IP-10, MCP-1, MIP-1α, MIP-1β, PDGF-BB, RANTES, TNF-α and VEGF) on a Luminex 100 System (BioRad, Hercules, CA, USA) according to the protocol provided by the manufacturer.

### Virological assessment

The virological assay protocol was performed as previously described [20], [35] with a cut-off value of 100 IU ml-1.

### Statistical analysis

All data were analyzed using SPSS 13.0 for Windows software (SPSS Inc., Chicago, IL, USA). Multiple comparisons were made among the different groups using the Kruskal-Wallis *H* non-parametric test. Comparisons between various individuals were performed using the Mann-Whitney *U* test; while comparisons between the same individual were performed using the Wilcoxon matched pairs *T* test. Correlations between variables were evaluated using the Spearman rank correlation test. For all tests, two-sided *P*<0.05 was considered to be significant.
